# Modeling the spread of COVID‐19 in New York City

**DOI:** 10.1111/pirs.12615

**Published:** 2021-06-28

**Authors:** Jose Olmo, Marcos Sanso‐Navarro

**Affiliations:** ^1^ Departamento de Análisis Económico Universidad de Zaragoza Spain; ^2^ Department of Economics University of Southampton United Kingdom

**Keywords:** Bayesian model averaging, COVID‐19, Poisson regression, prediction models, spatial effects

## Abstract

This paper proposes an ensemble predictor for the weekly increase in the number of confirmed COVID‐19 cases in the city of New York at zip code level. Within a Bayesian model averaging framework, the baseline is a Poisson regression for count data. The set of covariates includes autoregressive terms, spatial effects, and demographic and socioeconomic variables. Our results for the second wave of the coronavirus pandemic show that these regressors are more significant to predict the number of new confirmed cases as the pandemic unfolds. Both pointwise and interval forecasts exhibit strong predictive ability in‐sample and out‐of‐sample.

## Resumen

Este artículo propone un predictor de conjunto para el aumento semanal del número de casos confirmados de COVID‐19 en la ciudad de Nueva York a nivel de código postal. Dentro de un marco de promediación de modelo bayesiano, la línea de base es una regresión de Poisson para datos de recuento. El conjunto de covariables incluye términos autorregresivos, efectos espaciales y variables demográficas y socioeconómicas. Los resultados para la segunda ola de la pandemia de coronavirus muestran que estos regresores son más significativos para predecir el número de nuevos casos confirmados a medida que se desarrolla la pandemia. Tanto las previsiones puntuales como las de intervalo muestran una fuerte capacidad de predicción, tanto dentro como fuera de la muestra.

## 抄録

本稿は、ZIPコードレベルでのニューヨーク市におけるCOVID‐19の確定症例数の毎週の増加に対するアンサンブル予測因子を提案する。ベイズモデル平均化のフレームワーク内では、ベースラインはカウントデータのポアソン回帰である。共変量のセットには、自己回帰項、空間的影響、人口統計学的変数および社会経済的変数が含まれる。新型コロナウイルスのパンデミックの第2波に関する本稿の結果から、パンデミックが拡大するにつれて、これらのリグレッサーが新たに確認された症例数を予測する上でより有意であることが示される。点予測と間隔ごとの予測は、サンプル内とサンプル外の両方で強力な予測能力を示す。

## 1 INTRODUCTION

In the winter of 2019‐2020 the new SARS‐CoV‐2 virus started spreading from Wuhan (China), causing a new disease called COVID‐19 characterized by a virulent pneumonia and a high infection rate. The global impact of COVID‐19 has been profound, and the public health threat it represents is the most serious seen in a virus since the 1918 H1N1 influenza pandemic. Since 31 December 2019 and as of 28 February 2021, in accordance with the applied case definitions and testing strategies in the affected countries, more than 113 million confirmed cases of COVID‐19 have been reported to the World Health Organization (WHO), including 2,517,964 deaths.

The coronavirus pandemic has spread worldwide affecting, to a greater or lesser extent, most countries. It has hit hardest big cities such as Delhi, London, Madrid, Melbourne, New York, Paris or Rio de Janeiro. These cities are characterized not only by having a large number of inhabitants but also by being highly densely populated, owning a complex network of public transport, and accommodating large concentrations of workers in the services sector: education, healthcare, entertainment, retail, and finance. Individuals also suffer long commuting times to work, staggering income inequalities, and heterogenous living standards across districts, see Cheshire et al. (2014) and Nijman and Wei (2020). Urban agglomerations are also known to be very heterogeneous with respect to the composition of the population, including individuals from different races and cultural backgrounds (Shertzer & Walsh, 2019; Wei et al. 2018).

One of the cities that has been hit hardest by the coronavirus pandemic is New York (NYC). The impact of COVID‐19 in this city during the first wave that took place in the spring of 2020 has been widely studied in the literature, see Almagro et al. (2021), Almagro and Orane‐Hutchinson (2020), Borjas (2020), Glaeser et al. (2021), and Schmitt‐Grohé et al. (2020), among others. These important contributions focus on different aspects of the effects and transmission of the disease for the population of NYC, and generally consider data at zip code level. More specifically, Almagro and Orane‐Hutchinson (2020) explore different channels to explain the disparities in COVID‐19 incidence across NYC neighborhoods. These authors estimate several linear regression models to assess the statistical relevance of variables reflecting neighborhood characteristics and occupations, finding that the latter are important for explaining observed incidence patterns. Their results show that those occupations with a higher degree of human interaction are more likely to be exposed to the virus. A second contribution of Almagro and Orane‐Hutchinson (2020) is to suggest a selection on testing, whereby those residents in worse conditions are more likely to get tested, with such selection decreasing over time as tests become more widely available.

Borjas (2020) merges information on the number of tests and the number of infections at the zip code level with demographic and socioeconomic information from the decennial census and the American Community Survey. This author finds that people residing in poor or immigrant neighborhoods were less likely to be tested; but the likelihood that a test was positive was higher in these areas, as well as in those with larger households or predominantly black populations. The dependent variable in this study is the rate of infection in the population, which depends on both the frequency of tests and on the fraction of positive tests among those tested. One important contribution of Borjas (2020) is to show that the non‐randomness in testing across NYC neighborhoods partly invalidates standard statistical inferences between the rate of infection and the socioeconomic characteristics. Schmitt‐Grohé et al. (2020) investigate access to COVID‐19 testing across incomes using zip code level data on the number of tests, test results, and income per capita. These authors find that the distribution of tests across income levels is significantly more egalitarian than the distribution of income itself. Glaeser et al. (2020) analyze the efficiency of mobility restrictions in limiting COVID‐19 spread. Using zip code data for five U.S. cities, including New York, these authors find that total cases per capita decrease by 19*%* for every ten percentage point fall in mobility. Using panel data for NYC with week and zip code fixed effects, the decline increases to 30*%*.

This paper aims to extend the studies described above in several directions. First, we go beyond the spring of 2020, and analyze data on the number of COVID‐19 infections in NYC at zip code level from 1 September 2020 to 2 February 2021. Second, and most importantly, we focus on the prediction of the number of weekly new confirmed cases of the disease. Third, we include temporal and spatial effects in our set of explanatory variables. Temporal effects are captured by an autoregressive lag of the response variable and the lagged incidence rate, and spatial effects are accommodated by including their averages in contiguous neighborhoods. Fourth, we apply Bayesian model averaging (BMA) techniques using a generalized linear regression for count data as benchmark. The implementation of this methodology allows us to derive the posterior distribution of the parameters associated to the covariates. Thus, we shed light on the sensitivity of the increase in confirmed cases to demographic and socioeconomic factors, as well as autoregressive and spatial terms, under a potentially large number of specifications of the regression model. Fifth, we provide pointwise and interval forecasts for each week of the evaluation period and across NYC neighborhoods. By doing so, we model the uncertainty about our pointwise predictions. Finally, by fitting our empirical framework to cross‐sectional weekly data, we are able to accommodate the presence of a time‐varying intensity rate of the disease that is reflected in changes in the slope parameters. These dynamics can be associated to policy‐induced changes related to social distancing and the effect of the vaccine, among a few others, see Fernández‐Villaverde and Jones (2020).

The motivation of this paper is twofold. On the one hand, we acknowledge that standard linear models are not suitable for predicting the number of confirmed cases of the COVID‐19 disease as it is a count variable. To correct for this feature, we propose econometric frameworks that are more suitable for count data. In particular, our benchmark is a Poisson regression model with intensity parameter given by a linear function of a large set of demographic and socioeconomic variables, as well as temporal and spatial effects. On the other hand, we are not aware of econometric models for prediction of the COVID‐19 disease beyond Li and Linton (2021), Liu et al. (2021) and Fernández‐Villaverde and Jones (2020). Li and Linton (2021) fit a time series model based on a quadratic trend specification to country level data. Liu et al. (2021) use a panel data model to generate density forecasts for daily COVID‐19 infections for a sample of countries/regions. In this setup, the growth rate of active infections can be represented by autoregressive fluctuations around a downward sloping deterministic trend function with a break. Although Liu et al. (2021) also adopt a Bayesian approach, their methodology and objectives are very different from those in the present paper. Importantly, our model accommodates a large set of covariates, whereas these authors build on a stochastic version of the susceptible‐infected‐recovered (SIR) model of Kermack and McKendrick (1927, 1932, 1933) that incorporates dynamics in the model parameters and a structural break. Another recent important contribution focused on prediction that extends the SIR formulation is Fernández‐Villaverde and Jones (2020). These authors apply a standard epidemiological model to estimate and simulate the expansion of the coronavirus disease with data on deaths from several cities and countries around the world.

Two main conclusions can be drawn from our empirical analysis. First, the proposed set of covariates are more significant to predict the number of new COVID‐19 confirmed cases in NYC as the pandemic unfolds. Second, the factors with predictive power are different from those in the first wave. In contrast to income, household size, and occupational variables, we find that the persistence of the incidence rate, spatial effects, population size, and age‐related variables gain traction in this period compared to demographic and socioeconomic covariates. That is, the dynamic component of the proposed Poisson regression model is more relevant for prediction purposes than the static component given by the exogenous regressors reflecting neighborhood characteristics. The predictive performance of these models is assessed in‐sample and out‐of‐sample. Both Poisson regression and BMA frameworks report low mean square prediction errors from one‐period‐ahead pointwise forecasts. Interestingly, the baseline Poisson regression model reports very good fit in‐sample and out‐of‐sample, and its performance is comparable to the Bayesian ensemble predictor. In order to capture the uncertainty associated to the pointwise predictions, we also construct interval forecasts. The reliability and accuracy of these predictions is evaluated by computing empirical coverage probabilities at 90*%*. The information content of the intervals varies across periods but, in general, they are significantly narrower than during the first wave of the coronavirus pandemic, see Olmo and Sanso‐Navarro (2020). The autoregressive factors and the spatial effects seem to carry most of the predictive content compared to the spring of 2020. To illustrate further the reliability of our predictions, we use choropleth maps showing that our framework is able to capture the dynamics in the evolution of the pandemic and to predict the heterogeneity of new confirmed COVID‐19 cases across neighborhoods on a weekly basis.

The present paper is also related to the recent epidemiological studies by Cordes and Castro (2020) and DiMaggio et al. (2020). The first authors, in a descriptive analysis using zip code level data for NYC, identify areas with low access to testing and high case burden. Cordes and Castro (2020) analyze testing rates, positivity rates, and the proportion of positive tests, and relate them to socioeconomic variables. They find that clusters with less testing and low proportion of positive tests are characterized by higher income, education, and a dominant presence of white population. On the contrary, clusters with higher testing rates and shares of positive tests were disproportionately black and without health insurance. Simple correlation measures show inverse associations of the proportion of positive tests with white race, education, and income, and positive links with black race, Hispanic ethnicity, and poverty. DiMaggio et al. (2020) also claim that there is increasing evidence that, in addition to individual clinical factors, demographic, socioeconomic and racial characteristics of COVID‐19 infections play an important role. These authors analyze positive testing results counts within NYC neighborhoods with Bayesian hierarchical Poisson spatial models using integrated nested Laplace approximations. This study shows that spatial clustering accounts for approximately 32*%* of the variation in the data, with hot spots in all five boroughs. However, the strongest univariate association with positive testing rates is a clinical factor given by the proportion of residents with chronic obstructive pulmonary disease. 1

The rest of the paper is structured as follows. Section 2 discusses the dataset used for the study and describes the set of covariates. Section 3 introduces the forecast models, briefly reviewing Poisson regressions and BMA techniques for prediction. Section 4 shows the application of these methods to data from NYC at the zip code level. Suitable choices of variables with power to explain the cross‐sectional increase in the number of confirmed COVID‐19 cases are discussed, and a forecast evaluation exercise comparing the performance of several competing models is presented. Section 5 concludes. Tables and figures are collected in an appendix. A separate online appendix presents the results of the full analysis for all the weeks over the evaluation period.

## 2 DATA

**FIGURE 1:**
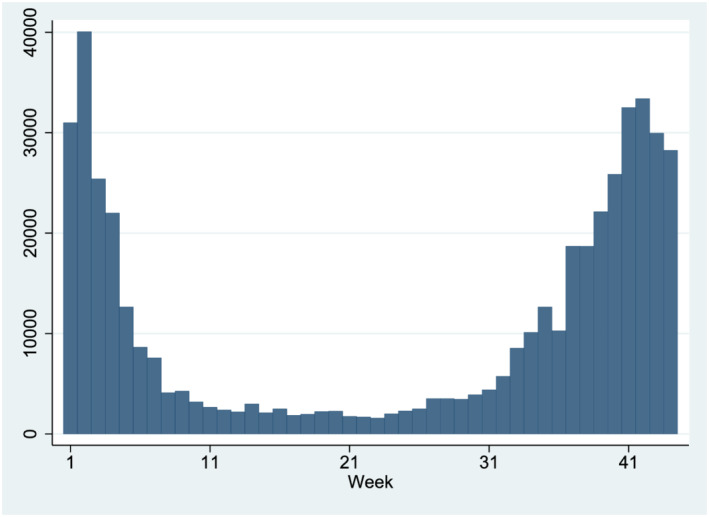
Weekly new confirmed COVID‐19 cases in New York City, 31 March 2020 – 2 February 2021

The NYC Department of Health and Mental Hygiene (DOH) compiles, since 31 March 2020, the cumulative count of confirmed COVID‐19 cases at the zip code level. 2 Although this information is available on a daily basis, and in line with most of the related studies discussed in the introductory section, the main variable of interest is the number of new confirmed COVID‐19 cases reported on a given week. Figure 1 displays the evolution of this variable in NYC until 2 February 2021. The peak of the first wave of the coronavirus pandemic took place in the second week of April, when the increase in the cumulative number of confirmed cases was 40,061, and the average number of cases per neighborhood reached 226. From that week onwards, this average number decreased steadily to 15 in the second of week of June, and remained below these levels until the beginning of September. After that moment, the number of weekly cases started to build up again reaching a maximum of 33,392 cases in the second week of January 2021.

This study exploits the information compiled during the second wave of the coronavirus pandemic in NYC, considering that it began in September 2020. Table 1 shows descriptive statistics of weekly new confirmed COVID‐19 cases in modified zip code tabulation areas (ZCTAs) on a 3‐week basis. In what follows, and for the sake of clarity, we are reporting the results using this frequency. The complete tables with the full set of results are shown in the online appendix. The figures reported in Table 1 reflect a steady increase in the cross‐sectional mean of new cases ranging from 11 in the second week of September 2020 to roughly 189 cases in the second week of January 2021. The dispersion in the number of cases has also increased during the period analyzed.

**TABLE 1:** Weekly new confirmed COVID‐19 cases in modified zip code tabulation areas (ZCTAs, N=177): Descriptive statistics

Period	Total	Mean	Standard deviation	Minimum	Maximum
8 Sep–15 Sep	1,992	11.254	10.251	0	66
29 Sep–6 Oct	3,520	19.887	25.033	0	187
20 Oct–27 Oct	3,891	21.983	16.340	0	79
10 Nov–17 Nov	8,533	48.209	32.627	0	164
1 Dec–8 Dec	10,277	58.062	63.942	0	400
22 Dec–29 Dec	22,123	124.989	87.499	6	435
12 Jan–19 Jan	33,392	188.655	126.137	3	624

The information of the demographic and socioeconomic characteristics of the neighborhoods have been extracted from the American Community Survey (ACS). This data correspond to 5‐year estimates for the period from 2014 to 2018. Table 2 describes the variables that have been used in our study. A first group of regressors reflects the importance of demographic factors in explaining the cross‐sectional variation of the increase in confirmed COVID‐19 cases at the zip code level, see Borjas (2020) and Schmitt‐Grohé et al. (2020). These variables are total population, its percentage of males, and the average household size. The shares of Blacks and African Americans, and of Hispanics or Latinos are intended to capture the racial composition of the population. The age structure is measured using the median age, the percentage of population 64 years and over, and the schooling enrollment rate.

**TABLE 2:** Weekly new confirmed COVID‐19 cases and potential determinants: Description of variables and data sources

Variable	Description	Sources
cases	Weekly new confirmed COVID‐19 cases	DOH
lcases	New confirmed cases during the previous week	DOH
lincid	Rate of new confirmed cases per 100,000 people during the previous week	DOH, ACS
lwcases	Average number of new confirmed cases during the previous week in contiguous ZCTAs	DOH
lwincid	Average rate of new confirmed cases per 100,000 people during the previous week in contiguous ZCTAs	DOH, ACS
popul	Total population	ACS
hhsize	Average household size	ACS
male	Percentage of male population	ACS
black	Black or African American, as per cent of total population	ACS
hispanic	Hispanic or Latino, as per cent of total population	ACS
age	Median age	ACS
over64	Percentage of population 65 years and over	ACS
enroll	Percentage of population 3 years and over enrolled in school	ACS
income	Per capita income in the past 12 months	ACS
selfemp	Percentage of households with self‐employment income	ACS
empl	Percentage of civilian noninstitutionalized population 18 to 64 years in the labor force that are employed	ACS
fulltime	Percentage of civilian noninstitutionalized population 18 to 64 years that worked full‐time, year round	ACS
pubtrans	Percentage of workers 16 years and over that use public transportation (excl. taxicab)	ACS
travtime	Mean travel time to work, in minutes	ACS
retail	Percentage of workers 16 years and over in retail trade	ACS
transp	Percentage of workers 16 years and over in transportation and warehousing, and utilities	ACS
eduheal	Percentage of workers 16 years and over in educational services, and health care and social assistance	ACS
entert	Percentage of workers 16 years and over in arts, entertainment, and recreation, and accommodation and food services	ACS
occup	Percentage of housing units that are occupied	ACS
rooms	Median number of rooms	ACS
internet	Percentage of households with an Internet subscription	ACS
nocov	Percentage of civilian noninstitutionalized population 19 to 64 years with no health insurance coverage	ACS

*Note:* Data sources are the American Community Survey, 5‐year estimates 2014‐2018 (ACS), and the New York City Department of Health and Mental Hygiene (DOH).

Economic conditions have been proxied by income per capita, the employment rate, the percentage of households with self‐employment income, and the share of full‐time workers. As pointed out by Harris (2020) and Hamidi and Hamidi (2021), public transport may have contributed to the spread of the coronavirus pandemic in NYC. We try to capture this influence by including as regressors the percentage of workers 16 years and over, the use of public transportation, excluding taxicab, and average travel time to work. Almagro and Orane‐Hutchinson (2020) find that occupations are important in explaining the differential incidence of COVID‐19 across NYC neighborhoods. For this reason, we have also considered the share of workers in sectors highly exposed to the virus as explanatory variables: retail trade; transportation, warehousing, and utilities; educational services, health care and social assistance; arts, entertainment, and recreation; and accommodation and food services. Living conditions have been reflected using the percentage of housing units that are occupied, the median number of rooms, the percentage of households with an Internet subscription, and the share of population lacking health insurance coverage.

The persistence in confirmed COVID‐19 cases is captured by two different variables: the number of new weekly confirmed cases and the incidence rate (ratio of confirmed cases per 100,000 inhabitants), both being lagged one period. These regressors are aimed at controlling for self‐reinforcing effects of the pandemic above and beyond the explanatory power of our set of demographic and socioeconomic variables. Spatial, neighboring, effects are also introduced into the model by including two additional regressors constructed as the weighted average of lagged new confirmed cases and of lagged incidence rates across contiguous locations. These spatial lags have been calculated using a row‐standardized contiguity spatial weights matrix, 3 constructed using the geographic information for modified ZCTAs provided by the U.S. Census Bureau.

## 3 METHODOLOGY

This section outlines the model used to describe the weekly evolution of the coronavirus pandemic in NYC neighborhoods. Standard regression models estimated using ordinary least squares (OLS) are not suitable in this context because they assume that the response variable is continuous. To apply an OLS estimation framework, the recent extant literature on the topic considers the incidence rate as the dependent variable, see Almagro and Orane‐Hutchinson (2020) and Borjas (2020) for linear regression and a group logit model, respectively. As an alternative, we directly model the number of weekly new confirmed cases. Therefore, as our response variable is count data, we consider as benchmark a generalized linear model (GLM) given by a Poisson regression. In order to accommodate a large set of potential covariates, we embed this specification within a BMA approach. It is characterized by an ensemble of predictors obtained from a battery of different Poisson regression models including different combinations of the covariates. This ensemble approach is known to reduce the forecast error and, more importantly, account for model uncertainty. In doing so, we are able to obtain posterior inclusion probabilities (PIPs) for each regressor, as well as posterior distributions for both the model parameters and the response variable.

### 3.1 Baseline model

The evolution of the number of new COVID‐19 cases in NYC at the zip code level is modeled as a Poisson regression, see McCullagh and Nelder (1989). This estimation framework allows us to use a count variable as response variable and model, directly, the increase in the number of confirmed cases per period. This variable is assumed to follow a Poisson distribution ℙ(λ), with *λ* denoting the intensity parameter. This function is convenient for modeling the expected number of arrivals of a random event for a fixed period of time, and is closely related to the exponential distribution that would model the interarrival times. In this setting, we assume that the number of new confirmed COVID‐19 cases is the number of *arrivals* to the neighborhood with confirmed cases from a given population, and *λ* is the average number of arrivals. Our predictions will be based on the estimates of this coefficient.

The Poisson regression model allows the intensity parameter to evolve over time and to depend on a set of *m* covariates. Mathematically, the number of confirmed cases per period is a Poisson random variable *y*_*it*_, with i=1,…,N, where *N* is the number of neighborhoods, and t=1,…,T, with *T* the number of periods. The expected value of this random variable is *λ*_*it*_, which we consider to depend on an intercept *α*_*t*_ and three types of regressors. The first one is an autoregressive dynamic component given by the lagged values of the number of new confirmed cases (*y*_*it* − 1_) and of the incidence rate (*z*_*it* − 1_). The second type of covariate contains a linear combination of *p* exogenous demographic and socioeconomic variables ${\left\{x_{ij}\right\}}_{j=1}^{p}$. These regressors are assumed to be constant over time because neighborhood characteristics are persistent variables that do not significantly change in the short run. As explained in the previous section, this information corresponds to the observational period 2014‐2018. The third type of covariate tries to capture spatial effects, and is defined using a contiguous adjacency matrix **W** that assigns ones to those zip codes that are contiguous and zeros, otherwise. For consistency with the spatial econometrics literature, we row‐standardize the matrix by normalizing the rows to sum to one. Therefore, the spatial lag of a given variable is its average value in contiguous neighborhoods. The model is as follows:

(1) $$\log\;\lambda_{it}=\alpha_{t}+\gamma_{t}y_{i,t-1}+\rho_{t}z_{i,t-1}+\sum_{j=1}^{m}\theta_{jt}x_{ij}+{\tilde{\gamma }}_{t}w_{i}y_{t-1}+{\tilde{\rho }}_{t}w_{i}z_{t-1},$$

with *γ*_*t*_ and *ρ*_*t*_ are the autoregressive parameters associated to the dynamic component; *θ*_*jt*_ are the parameters corresponding to each regressor j=1,…,p; ${\tilde{\rho }}_{t}$ and ${\tilde{\gamma }}_{t}$ are the parameters capturing the spatial effects; *w*_*i*_ is a row vector of the row‐standardized contiguity spatial weights matrix **W**; $z_{t-1}={\left(z_{1,t-1},\ldots ,z_{N,t-1}\right)}^{\prime }$, and $y_{t-1}={\left(y_{1,t-1},\ldots ,y_{N,t-1}\right)}^{\prime }$. The econometric specification of the spatial effects in model (1) is reminiscent of spatial autoregressive (SAR) model, see Anselin (2003); however, in contrast to this model, we avoid the presence of endogeneity by considering the spatial terms to be lagged one period. This specification is plausible in our dynamic context and convenient for predictive purposes.

### 3.2 Bayesian model averaging

Model averaging techniques – available both in frequentist and Bayesian contexts – consist of estimating all candidate models and then computing a weighted average of their estimates, taking into account the implicit uncertainty conditional on a given model and across different models. This approach was originally developed for linear regression models, see Raftery (1995) and Raftery et al. (1997), and subsequently extended to more general frameworks. An early contribution to BMA with GLMs is Raftery (1996), who proposes to use approximations for the Bayes factors, based on the Laplace method for integrals. This author also suggests a way to elicit reasonable, but data‐dependent, proper priors.

In general, BMA assigns a prior probability to a set of models, and to a set of parameters associated to each model. These prior distributions are updated once we incorporate the information obtained from the data to obtain the posterior distributions. Therefore, BMA is able to deal simultaneously with model selection, estimation, and inference. There are $K=2^{m}$ models comprised by all possible combinations of the *m* covariates in the Poisson regression (1). Each model is denoted as *M*_*k*_, with k=1,…,K, and depends on a vector of parameters $\beta_{k}$ with a conditional posterior probability given by:

(2) $$g(\beta_{k}\;|\;y,M_{k})=\frac{f(y\;|\;\beta_{k},M_{k})g(\beta_{k}\;|\;M_{k})}{f(y\;|\;M_{k})},$$

where $f(y\;|\;\beta_{k},M_{k})$ and $g(\beta_{k}\;|\;M_{k})$ denote, respectively, the likelihood function of the observations, and the prior density function of the parameters, conditional on model *M*_*k*_. The literature on BMA models has studied extensively the suitability of prior density functions. Reviews of the most popular options, both in linear and GLM frameworks, can be found in Forte et al. (2018), Li and Clyde (2018), and Steel (2020).

For a given prior model probability *P*(*M*_*k*_), its posterior probability can be calculated applying Bayes' rule:

(3) $$P(M_{k}\;|\;y)=\frac{f(y\;|\;M_{k})P(M_{k})}{f(y)},$$

with *f*(**y** | *M*_*k*_) and *f*(**y**) the conditional and marginal likelihood functions, respectively. Leamer (1978) assumes that the true parameters β associated to the regression variables are a function of $\beta_{k}$, from a specific model *M*_*k*_. This author proposes to obtain the posterior density function of β, conditional on the data ${\left\{y_{i}\right\}}_{i=1}^{N}$ and model candidates ${\left\{M_{k}\right\}}_{k=1}^{K}$, using the law of total probability:

(4) $$g(\beta \;|\;y)=\sum_{k=1}^{K}P(M_{k}\;|\;y)g(\beta_{k}\;|\;y,M_{k}).$$

When *m* is moderate to large, posterior probabilities of individual models can be very small and their interpretation loses appeal. In such situations, inclusion probabilities are very useful. Formally, the PIP for variable *x*_*j*_, with j=1,…,m, is:

(5) $$P(d_{j}=1\;|\;\text{y}\;)=\sum_{k=1}^{K}P(d_{k}=1\;|\;M_{k},\text{y}\;)P(M_{k}\;|\;\text{y})=\overset{K}{\underset{x_{k}\in M_{k}}{\underset{k=1}{\sum }}}\in M_{k}^{K}P(M_{k}\;|\;\text{y}),$$

where $d_{j}=1$ if variable *j* is included in the model, zero otherwise. Using these models we can obtain the predictive distribution of the quantity of interest. For the response variable **y**, this distribution, denoted as $\hat{\text{y}}$, is computed as:

(6) $$p(\hat{\text{y}}\;|\;\text{y}\;)=\sum_{k=1}^{K}\left[\int_{B_{k}}p(\hat{\text{y}}\;|\;\beta_{k},M_{k},\text{y}\;)g(\beta_{k}\;|\;M_{k},\text{y}\;)d\beta_{k}\right]P(M_{k}\;|\;\text{y}),$$

where the quantity in square brackets is the predictive distribution given *M*_*k*_, obtained using the posterior density function defined in (2). This function is integrated over the space of parameters $\beta_{k}\in B_{k}$, for each k=1,…,K. In practice, the BMA approach can be simplified using Monte‐Carlo simulation methods or algorithms to rule out models with low probability of generating the observations such as the Occam's window, see Madigan and Raftery (1994) and Raftery et al. (1997), among others.

## 4 COVID‐19 IN NYC NEIGHBORHOODS

This section presents the findings of our empirical study for weekly data on the number of new confirmed COVID‐19 cases in NYC. We divide the analysis into two exercises. First, we discuss model selection and focus on the relevance of the set of covariates presented in Section 2 to explain the differential evolution of the coronavirus pandemic across neighborhoods. Second, we assess the predictive power of these variables both in‐sample and out‐of‐sample.

### 4.1 Poisson regression

Table 3 reports the estimation results of the Poisson regression model (1) for selected weeks in our sample period separately. 4 The variables that exhibit statistical significance across most periods are the dynamic terms given by increase in the number of confirmed cases and the incidence rate in the previous week. Both variables display a positive coefficient and gain statistical significance as the number of cases accelerates through the wave. The spatial terms are also significant in most periods, highlighting the influence of neighboring ZCTAs in the evolution of the pandemic. As can be observed in Table A1 of the online appendix, this effect is particularly important in the last two weeks of the sample period analyzed when the pandemic gains traction. This finding also suggests that the presence of positive spillovers in the spread of COVID‐19 across neighborhoods helps to predict the evolution of the disease in NYC.

**TABLE 3:** Poisson regression: Weekly new confirmed COVID‐19 cases

	8 Sep–15 Sep	29 Sep–6 Oct	20 Oct–27 Oct	10 Nov–17 Nov	1 Dec–8 Dec	22 Dec–29 Dec	12 Jan–19 Jan
lcases	0.033***	‐0.005*	0.003	0.005***	0.005***	0.003***	3.46e‐04*
	(0.007)	(0.003)	(0.003)	(0.001)	(5.02e‐04)	(2.91e‐04)	(1.76e‐04)
lincid	‐0.004	0.014***	0.002	0.001***	0.001***	‐3.74e‐05	0.001***
	(0.004)	(0.002)	(0.002)	(3.54e‐04)	(02.62e‐04)	(1.50e‐04)	(1.18e‐04)
lwcases	‐0.009	0.002	0.008***	‐0.002**	0.003***	0.001***	3.78e‐04***
	(0.008)	(0.004)	(0.002)	(0.001)	(4.43e‐04)	(1.85e‐04)	(1.03e‐04)
lwincid	0.021***	0.001	‐0.007**	0.005***	4.09e‐04	4.63e‐04***	2.66e‐04**
	(0.006)	(0.004)	(0.003)	(0.001)	(2.86e‐04)	(1.43e‐04)	(1.06e‐04)
popul	1.49e‐05***	1.38e‐05***	1.75e‐05***	1.15e‐05***	9.91e‐06***	7.96e‐06***	1.18e‐05***
	(3.03e‐06)	(2.44e‐06)	(2.28e‐06)	(1.54e‐06)	(1.40e‐06)	(9.27e‐07)	(9.51e‐07)
hhsize	‐0.839***	‐0.176	‐0.322***	‐0.279***	‐0.583***	‐0.066	‐0.096***
	(0.148)	(0.114)	(0.104)	(0.074)	(0.068)	(0.044)	(0.035)
male	‐0.041**	‐0.014	0.018*	‐0.012	0.035***	‐0.005	‐0.002
	(0.017)	(0.013)	(0.011)	(0.008)	(0.007)	(0.005)	(0.004)
black	‐0.003	‐0.006***	‐0.008***	‐0.006***	‐0.015***	‐0.005**	‐0.001**
	(0.002)	(0.002)	(0.002)	(0.001)	(0.001)	(0.001)	(0.001)
hispanic	0.006**	‐0.001	0.001	0.004***	‐0.005***	0.001*	2.40e‐04
	(0.002)	(0.002)	(0.002)	(0.001)	(0.001)	(0.001)	(5.49e‐04)
age	‐0.045***	‐0.064***	‐0.037***	‐0.023***	‐0.069***	‐0.017***	‐0.018***
	(0.014)	(0.011)	(0.010)	(0.007)	(0.006)	(0.004)	(0.003)
over64	‐1.04e‐05	4.49e‐05***	2.56e‐05*	2.75e‐05***	‐6.55e‐07	2.58e‐05***	3.69e‐05***
	(1.98e‐05)	(1.59e‐05)	(1.40e‐05)	(9.02e‐06)	(9.01e‐06)	(6.28e‐06)	(4.74e‐06)
enroll	‐0.002	‐0.016*	‐0.008	‐0.007	‐0.065***	‐0.019***	‐0.004
	(0.012)	(0.010)	(0.008)	(0.006)	(0.006)	(0.004)	(0.003)
income	‐9.47e‐06***	‐5.95e‐06**	2.41e‐06	‐1.06e‐06	‐2.23e‐06	‐2.24e‐06**	‐1.05e‐06
	(3.31e‐06)	(2.62e‐06)	(2.19e‐06)	(1.51e‐06)	(1.51e‐06)	(1.01e‐06)	(8.28e‐07)
selfemp	0.045***	0.029***	0.010	0.034***	‐0.011**	0.007**	0.010***
	(0.011)	(0.009)	(0.008)	(0.005)	(0.005)	(0.003)	(0.003)
empl	0.026	0.027**	‐0.007	0.032***	‐0.023***	‐0.001	‐0.004
	(0.016)	(0.013)	(0.012)	(0.008)	(0.007)	(0.005)	(0.0045)
fulltime	0.003	‐0.003	‐1.93e‐04	0.006	‐0.007*	0.001	0.002
	(0.010)	(0.008)	(0.007)	(0.005)	(0.004)	(0.003)	(0.002)
pubtrans	‐0.004	‐0.006*	‐0.006**	3.46e‐04	0.022***	‐2.42e‐04	‐0.003***
	(0.004)	(0.003)	(0.003)	(0.002)	(0.002)	(0.001)	(0.001)
travtime	‐4.70e‐06	6.16e‐06	‐4.51e‐05	3.30e‐05	‐3.77e‐04***	1.79e‐05	6.99e‐05***
	(9.38e‐05)	(7.58e‐05)	(7.17e‐05)	(4.75e‐05)	(4.85e‐05)	(3.06e‐05)	(2.46e‐05)
retail	0.015	0.012	0.032**	0.050***	0.018**	‐0.001	0.003
	(0.018)	(0.015)	(0.013)	(0.009)	(0.008)	(0.006)	(0.004)
transp	0.056***	0.034**	0.066***	0.041***	0.132***	0.034***	0.039***
	(0.017)	(0.014)	(0.012)	(0.008)	(0.008)	(0.005)	(0.004)
eduheal	0.018*	0.031***	0.011	0.025***	0.044***	0.013***	0.006**
	(0.010)	(0.008)	(0.007)	(0.005)	(0.004)	(0.003)	(0.002)
entert	‐0.016	0.036***	0.036***	0.023***	‐0.008	0.009**	0.015***
	(0.015)	(0.012)	(0.010)	(0.007)	(0.006)	(0.004)	(0.003)
occup	0.005	0.002	0.002	‐0.005	‐0.009**	0.004	0.015***
	(0.008)	(0.006)	(0.005)	(0.003)	(0.004)	(0.003)	(0.002)
rooms	0.203***	0.039	0.103*	0.157***	0.602***	0.113***	‐0.023
	(0.073)	(0.060)	(0.053)	(0.036)	(0.035)	(0.023)	(0.019)
internet	0.009	0.004	‐0.001	‐0.009***	‐0.013***	‐0.010***	‐0.002
	(0.007)	(0.006)	(0.005)	(0.003)	(0.003)	(0.002)	(0.002)
nocov	‐0.003	‐0.021***	‐0.018***	‐0.006	‐0.016***	‐0.001	‐7.84e‐06
	(0.010)	(0.007)	(0.007)	(0.004)	(0.004)	(0.003)	(0.002)
constant	1.541	1.320	2.843**	‐0.084	7.737***	4.516***	3.092***
	(1.950)	(1.602)	(1.372)	(0.921)	(0.871)	(0.606)	(0.468)
Log likelihood	‐601.626	‐589.701	‐597.097	‐764.797	‐2,741.613	‐926.591	‐1,064.428

*Note:* The number of observations is 177. Standard errors in parentheses. *** *p* < 0.01, ** *p* < 0.05, * *p* < 0.10.

Population size is strongly significant and displays a positive relationship with the response variable. This result is not surprising because total population can be considered to proxy for other fundamental factors reflecting living standards, population density, and deprivation levels. In contrast to the findings reported for the first wave (Almagro et al. 2021; Olmo & Sanso‐Navarro, 2020), household size is negatively related to the number of new confirmed cases, but the magnitude of the parameter decreases over time. It is worth noting that contagion at the household level may be captured by the median number of rooms. The percentage of male population is hardly significant during our sample period. Interestingly, and in clear contrast to the results obtained for the spring of 2020 (Choi, 2020), the racial composition of the neighborhoods does not have a prominent role in explaining differences in the number of new confirmed cases across zip codes during the second wave.

Age‐related variables have a strong predictive power in this model specification. The median age of the population is negatively associated to the number of weekly COVID‐19 cases. Nonetheless, those neighborhoods with a large proportion of people over 64 years old tend to exhibit a higher number of new confirmed cases. Whereas age may be a proxy for other socioeconomic variables such as income, the share of the elderly reflects the strong influence of the coronavirus pandemic on this age group. During the first wave, income and the number of cases had a negative relationship that was highly significant, see Olmo and Sanso‐Navarro (2020). However, income per capita is hardly significant in our sample period, suggesting that the number of infections is not very related to the income of the neighborhood. Similarly, and in contrast to existing findings for the spring of 2020, occupational variables lose most of their significance. The main exceptions are the self‐employment rate and some variables reflecting the sectoral composition of employment. In particular, those measuring the importance of the transport, education and health, and entertainment sectors. In all these three cases the effects are quite significant, reflecting that those neighborhoods with a large fraction of individuals working on these sectors are more affected by the disease. Access to Intern et also has an effect during the last weeks of the study that, as expected, is negative. Other variables such as average commuting time, and having a full‐time job lose any statistical significance during this second wave of the coronavirus pandemic.

### 4.2 Bayesian model averaging

The estimation results of the Poisson regressions displayed in Table 3 may vary if only a subset of the covariates is included. For this reason, and in order to control for model uncertainty, the same analysis has been carried out adopting a BMA approach. 5 That is to say, we do not impose any specification a priori and let data speak. The sampling method combines a random walk Metropolis‐Hastings algorithm with a random swap between included and excluded variables, see Raftery et al. (1997) and Clyde et al. (2011), with one million iterations. We have specified 6 a uniform prior over the model space, and a benchmark prior – which displays a good performance for both estimation and prediction purposes – for model‐specific parameters (Li & Clyde, 2018). In addition to the implementation of BMA, we use the posterior means under model selection. This corresponds to a decision rule that combines estimation and selection. In particular, we consider the highest probability (HPM) and the best prediction (BPM) models. From a Bayesian decision theory perspective, the latter is the model that is closest to BMA predictions under squared error loss. Liang et al. (2008) discuss these alternative Bayesian methods in some detail.

Table 4 reports the PIP (5) for each covariate in selected periods. This analysis complements the preceding one on the statistical significance of the regressors based on t‐tests. In this case, the relevance of the covariates in the ensemble predictor relies on the probability that they are included in the model specification. The discussion of the results can be separated by the type of covariate. The inclusion probabilities associated to the temporal lag of the response variable are very low in most periods, suggesting that this term does not contain much predictive power. In contrast, the lagged incidence rate receives a PIP close to one in most periods. The variables that capture the spatial effects are very significant, what corroborates their importance for prediction purposes. The racial composition and age‐related variables are other important factors that are picked up by the ensemble predictor. On the contrary, variables related to labor market conditions are hardly relevant in this setting. The predictive performance of the variables reflecting the sectoral composition of employment is mixed. During the first weeks of our sample period these regressors receive very low PIPs, however, during the last weeks, most of these variables achieve very high values of these probabilities. This is particularly the case of the percentages of workers in the transport, education and health, and entertainment sectors.

**TABLE 4:** Bayesian model averaging: Posterior inclusion probabilities

	8 Sep–15 Sep	29 Sep–6 Oct	20 Oct–27 Oct	10 Nov–17 Nov	1 Dec–8 Dec	22 Dec–29 Dec	12 Jan–19 Jan
lcases	0.274	1.000	0.064	0.090	1.000	0.055	0.067
lincid	0.999	1.000	0.993	0.980	0.103	1.000	1.000
lwcases	0.038	0.377	0.975	0.274	1.000	0.992	1.000
lwincid	0.040	0.244	0.809	1.000	1.000	1.000	0.543
popul	0.181	1.000	0.999	1.000	0.999	1.000	1.000
hhsize	0.158	0.053	0.084	0.141	0.105	0.055	0.292
male	0.066	0.043	0.959	0.128	0.053	0.244	0.162
black	0.990	1.000	0.638	0.467	0.808	1.000	0.982
hispanic	0.189	1.000	0.121	0.719	0.917	0.057	0.621
age	0.926	1.000	0.680	0.093	0.985	0.999	1.000
over64	0.999	0.992	0.846	0.076	1.000	1.000	1.000
enroll	0.044	0.091	0.121	0.272	0.475	0.973	0.990
income	0.993	0.894	0.084	0.910	0.218	0.214	0.046
selfemp	0.151	0.080	0.332	0.719	0.440	0.113	0.094
empl	0.045	0.037	0.095	0.152	0.042	0.676	0.909
fulltime	0.048	0.264	0.139	0.246	0.083	0.149	0.061
pubtrans	0.122	0.072	0.105	0.064	0.767	0.571	0.046
travtime	0.047	0.109	0.066	0.749	0.832	0.110	0.164
retail	0.041	0.041	0.164	0.039	0.042	0.475	0.985
transp	0.049	0.074	0.699	0.052	0.998	1.000	1.000
eduheal	0.093	0.998	0.862	0.089	0.832	0.998	0.058
entert	0.052	0.083	0.261	0.347	0.861	1.000	1.000
occup	0.038	0.095	0.047	0.040	0.122	0.043	1.000
rooms	0.055	0.335	0.102	0.049	0.359	0.087	0.092
internet	0.041	0.147	0.078	0.065	0.339	0.266	0.051
nocov	0.052	0.043	0.095	0.996	0.057	0.462	1.000

*Note:* The number of observations is 177. The sampling method consists of a random walk Metropolis‐Hastings algorithm combined with a random swap. A benchmark and uniform priors have been established, respectively, for parameters and models.

Two main conclusions can be drawn from this analysis. On the one hand, the inclusion probabilities increase over time as the pandemic unfolds, implying that the covariates are more significant to predict the number of new confirmed cases at the end of the sample period. On the other hand, the factors with predictive power are different from those in the first wave when the self‐employment rate and income per capita were important covariates. During the second wave of the coronavirus pandemic these variables lose their predictive power. Instead, we find that the persistence of the incidence rate, captured by its first lag, the spatial effects, population size, and age‐related variables gain traction compared to socioeconomic variables. That is, the dynamic component of the Poisson regression model is more relevant for prediction purposes than the static component given by the exogenous regressors.

**FIGURE 2:**
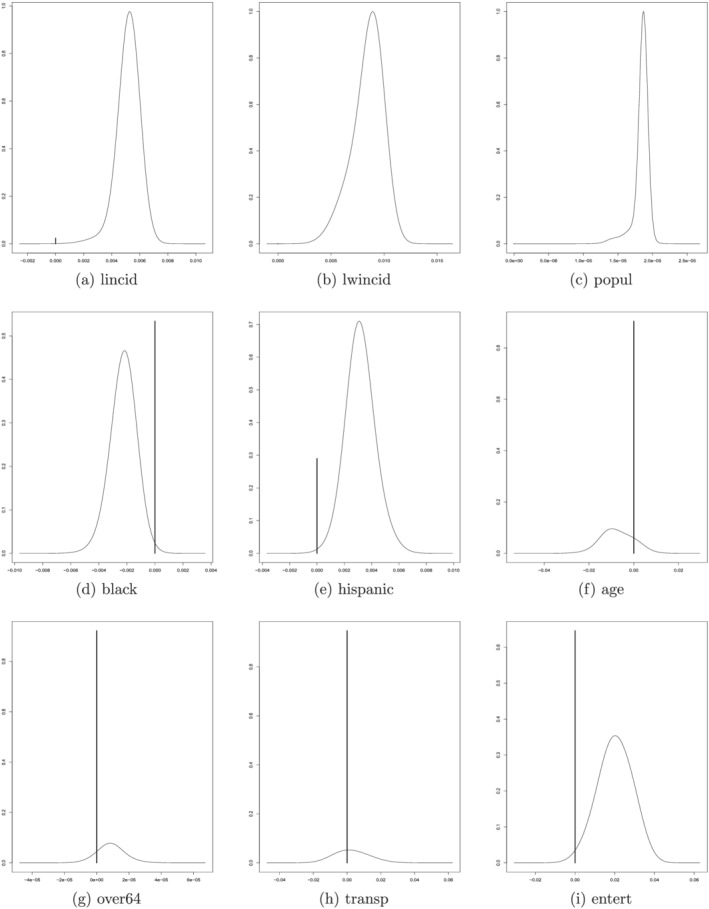
Bayesian model averaging: Posterior density functions for the coefficients of selected regressors, 12 Jan – 19 Jan

To gain a better insight into the nature of the relationship between the predictors and the response variable with the BMA model, we plot in Figure 2 the posterior density functions of the model parameters (4) associated to a selection of regressors, according to their high PIPs during the sample period analyzed, for the second week of January 2021, when the peak of the second wave was reached. The density functions, from left to right and then going down, correspond to the lagged incidence rate, its average over contiguous neighborhoods, total population, the racial composition (black and hispanic), median age, the share of the elderly, and the percentages of workers in the transport and entertainment sectors. The plots are very informative about the sign of the relationship between these variables and the increase in the number of confirmed COVID‐19 cases. The density functions also show the presence of uncertainty about the parameters associated to each regressor. The probability that the covariates are not correlated to the response variable, characterized by β=0, is represented by the height of a vertical line. These density functions reinforce the previous findings: the incidence rate, the spatial effects of contiguous neighborhoods, and population size are strongly significant predictors in the BMA specification. In contrast, the other variables are far less relevant.

### 4.3 Predictive performance

This subsection carries out an exercise to assess the predictive performance of the models implemented above. We consider five competing forecast methods. The first one is based on a Poisson random variable with intensity parameter given by the cross‐sectional sample average ${\bar{y}}_{t}$. This model corresponds to fitting the data on the number of infections in each period to a Poisson regression model using maximum likelihood. The second model is the Poisson regression with intensity parameter introduced in (1). The remaining three methods are different versions of the BMA approach, discussed before: a pure model averaging (BMA), the model that receives the highest probability (HPM), and the model with the best predictive performance (BPM).

The loss function that is used to evaluate the in‐sample predictions of the models is the root mean square error, calculated as $RMSE_{t}=N^{-1/2}{\left[\sum_{i=1}^{N}{\left(y_{it}-{\hat{y}}_{it}\right)}^{2}\right]}^{1/2}$, with *y*_*it*_ the realized number of new confirmed cases in each neighborhood i=1,…,N for a given time period t=1,…,T; and ${\hat{y}}_{it}$ the associated predictions obtained from the different methods. Similarly, the performance of the out‐of‐sample predictions is assessed with the root mean square prediction error, obtained as $RMSPE_{t+1|t}=N^{-1/2}{\left[\sum_{i=1}^{N}{\left(y_{i,t+1}-{\hat{y}}_{it+1}\right)}^{2}\right]}^{1/2}$, with *y*_*i*, *t* + 1_ the realized number of weekly new cases in each neighborhood i=1,…,N at period *t* + 1. The in‐sample predictions are obtained with data from period *t* − 1 and tested with the data in *t*; whereas the out‐of‐sample predictions are obtained combining previous estimated parameters with data from period *t* and tested with data from period *t* + 1. For completeness, we also report the relative counterparts of the RMSE and RMSPE statistics, obtained from a loss function defined for the prediction error over the realized observation. For the in‐sample exercise we have $RMSE_{t}^{r}=N^{-1/2}{\left\{\sum_{i=1}^{N}{\left[\left(y_{it}-{\hat{y}}_{it}\right)/y_{it}\right]}^{2}\right\}}^{1/2}$, and for the out‐of‐sample predictions we obtain $RMSPE_{t+1|t}^{r}=N^{-1/2}{\left\{\sum_{i=1}^{N}{\left[\left(y_{i,t+1}-{\hat{y}}_{it+1}\right)/y_{i,t+1}\right]}^{2}\right\}}^{1/2}$.

There is a well‐established literature indicating the predictive advantages of ensemble predictions with respect to simple specifications of the data. Madigan and Raftery (1994) state that BMA methods predict at least as well as any single model in terms of logarithmic probability score. (Min & Zellner, 1993) show that the expected squared error loss of point forecasts is always minimized by BMA, provided the model space includes the one that generated the data. These important results highlight the superiority of BMA predictions in linear settings. Nonetheless, our problem concerns the predictions of a GLM for count data. In this setting it is not clear that the findings of the above authors naturally carry forward to ensembles of Poisson regression models. Therefore, the empirical forecasting evaluation exercise that we implement in this subsection is of independent value.

Table 5 presents both RMSE and RMSPE statistics for the five competing forecast methods. The comparison of the loss functions across them suggests that the naive Poisson regression model performs worse than the other competitors. By contrast, the predictions of the other four methods are comparable in‐sample in terms of RMSE and out‐of‐sample in terms of RMSPE. The Poisson regression model shows a very good performance compared to the three Bayesian methods. In particular, the predictions of the Poisson regression are comparable to, and in some periods even outperform, those of the other three ensemble predictors. This is the case for all periods except the week starting on 12 January 2021, when the number of infections reaches a peak. These empirical findings are robust across evaluation periods, and for the in‐sample and out‐of‐sample exercises.

**TABLE 5:** Predictive performance: Root mean square errors

Relative, in‐sample							
	8 Sep–15 Sep	29 Sep–6 Oct	20 Oct–27 Oct	10 Nov–17 Nov	1 Dec–8 Dec	22 Dec–29 Dec	12 Jan–19 Jan
Benchmark	542.735	942.967	766.300	1136.260	637.715	565.335	602.540
Poisson	1.068	1.094	0.886	1.064	1.145	0.364	0.558
BMA	1.080	1.205	0.951	1.147	1.206	0.381	0.538
HPM	1.093	1.243	0.928	1.149	1.204	0.386	0.530
BPM	1.102	1.213	0.922	1.141	1.224	0.377	0.535

Relative, out‐of‐sample							
	8 Sep–15 Sep	29 Sep–6 Oct	20 Oct–27 Oct	10 Nov–17 Nov	1 Dec–8 Dec	22 Dec–29 Dec	12 Jan–19 Jan
Benchmark	495.856	509.491	708.343	385.556	414.464	1982.334	1184.900
Poisson	1.326	0.624	0.672	0.697	4.145	0.558	1.208
BMA	1.380	0.712	0.848	1.670	4.197	0.614	1.166
HPM	1.420	0.729	0.813	2.250	4.142	0.610	1.166
BPM	1.399	0.731	0.804	1.572	4.241	0.604	1.166

Absolute, in‐sample							
	8 Sep–15 Sep	29 Sep–6 Oct	20 Oct–27 Oct	10 Nov–17 Nov	1 Dec–8 Dec	22 Dec–29 Dec	12 Jan–19 Jan
Benchmark	10.251	25.033	16.340	32.627	63.942	87.499	126.137
Poisson	4.781	7.629	6.365	10.948	16.622	21.571	31.966
BMA	4.829	7.900	6.507	11.190	16.545	21.763	32.423
HPM	4.889	7.989	6.530	11.320	16.697	21.983	32.744
BPM	4.872	7.986	6.548	11.208	16.842	21.981	32.662

Absolute, out‐of‐sample							
	8 Sep–15 Sep	29 Sep–6 Oct	20 Oct–27 Oct	10 Nov–17 Nov	1 Dec–8 Dec	22 Dec–29 Dec	12 Jan–19 Jan
Benchmark	10.396	25.033	16.593	33.838	79.714	89.992	127.625
Poisson	8.907	10.101	9.320	17.926	59.494	34.309	55.450
BMA	9.248	10.013	9.274	33.520	60.378	34.886	55.294
HPM	9.286	10.147	9.337	44.262	59.464	34.768	56.040
BPM	9.178	10.299	9.317	31.931	60.705	34.740	55.403

The second main conclusion derived from the results reported in Table 5 is about the accuracy of the predictions. The magnitude of the loss functions for the absolute RMSE and RMSPE is smaller than that of the response variable. As it can be observed in Table 1, the average number of new infections across neighborhoods during the first week of our sample period is 11.254. The in‐sample predictions for that week yield a RMSE ranging between 4.781 and 4.889, suggesting a very good in‐sample fit. The predictive performance is not as good for the corresponding out‐of‐sample exercise at the bottom panel of Table 5. Nevertheless, the results of the loss function across models show the value of the model predictions. The performance of the different methods improves in the second period, when the average number of infections is 19.887 and the in‐sample RMSE is between 7.629 and 7.900. The corresponding RMSPE is around 10, which shows the ability of the models to predict the number of cases one period ahead.

Similar results are obtained as we move along the pandemic wave. The loss functions are steadily increasing, what reflects the steep rise in the number of infections across neighborhoods. In the last week under evaluation the average number of positive cases is 188.655. The in‐sample RMSE ranges between 31.966 and 32.744, whereas the out‐of‐sample RMSE ranges between 55.294 and 56.040. These figures show that the four prediction models are able to capture the positive trend in the number of infections. Importantly, the comparison of the loss functions with the naive Poisson regression model also highlights the value of incorporating the covariates in the model specification. The model that simply fits the count data to the number of new confirmed cases reports a RMSPE of 127.625. However, the models that exploit the information contained in autoregressive and spatial terms, as well as in demographic and socioeconomic variables, reduce the loss function by half.

The empirical loss functions can also be used to construct confidence intervals for the pointwise predictions of each model. The intervals of the in‐sample predictions are calculated as ${\hat{y}}_{it}\pm z_{1-\alpha /2}RMSE_{t}$, with *z*_1 − *α*/2_ being the critical value of a standard normal distribution for an *α* significance level, and a sample size *N* sufficiently large. Similarly, the predictive intervals for the out‐of‐sample exercise are constructed as ${\hat{y}}_{it+1}\pm z_{1-\alpha /2}RMSPE_{t+1|t}$. To assess the accuracy of these intervals in capturing the actual observations we calculate their corresponding empirical coverage probability. This quantity is obtained as the fraction of the number of observations *y*_*it*_, for a given *t*, that lie inside the interval forecasts. More formally, the empirical coverage probability for the in‐sample prediction analysis is defined as $N^{-1}\overset{N}{\underset{i=1}{\sum }}1\left(|y_{it}-{\hat{y}}_{it}|\leq z_{1-\alpha /2}RMSE_{t}\right)$, with 1(·) an indicator function that takes a value of one if the argument is true, zero otherwise. The out‐of‐sample empirical coverage probability is $N^{-1}\overset{N}{\underset{i=1}{\sum }}1\left(|y_{i,t+1}-{\hat{y}}_{it+1}|\leq z_{1-\alpha /2}RMSPE_{t+1|t}\right)$.

Table 6 reports the empirical coverage probabilities obtained for predictive intervals constructed with a 90*%* confidence. These figures show empirical coverages that are close to the nominal ones across methods and periods. The accuracy of the intervals slightly varies between the in‐sample and out‐of‐sample exercises, and also across periods, but not much across methods. As expected, the empirical coverage probability of the predictive intervals in‐sample is closer to the nominal one than that for those out‐of‐sample. Overall, the analysis in Table 6 presents a very satisfactory performance of the predictive intervals for the Poisson regression and the three Bayesian models. The only exceptions are the BMA methods during the week starting on 10 November, 2020 which present an inflated coverage probability. The good performance of the interval forecasts allows us to interpret the RMSE and RMSPE as reliable estimators of their width. For example, the interval forecast for the average value associated to the Poisson regression model for the in‐sample exercise for the week starting on 8 September 2020 is (3.41, 19.09). This interval is obtained as 11.254 ± 1.64 × 4.781. Similarly, the corresponding interval forecast for the last week reported in Table 5 is (136.20, 241.07), calculated as 188.655 ± 1.64 × 31.966.

**TABLE 6:** Predictive performance: Empirical coverage probabilities

In‐sample							
	8 Sep–15 Sep	29 Sep–6 Oct	20 Oct–27 Oct	10 Nov–17 Nov	1 Dec–8 Dec	22 Dec–29 Dec	12 Jan–19 Jan
Benchmark	0.96	0.960	0.938	0.977	0.96	0.955	0.932
Poisson	0.915	0.926	0.904	0.910	0.915	0.915	0.887
BMA	0.926	0.910	0.910	0.904	0.910	0.915	0.887
HPM	0.921	0.915	0.915	0.898	0.910	0.921	0.881
BPM	0.921	0.910	0.910	0.915	0.921	0.932	0.893

Out‐of‐sample							
	8 Sep–15 Sep	29 Sep–6 Oct	20 Oct–27 Oct	10 Nov–17 Nov	1 Dec–8 Dec	22 Dec–29 Dec	12 Jan–19 Jan
Benchmark	0.949	0.943	0.915	0.932	0.955	0.926	0.921
Poisson	0.938	0.915	0.887	0.932	0.910	0.904	0.910
BMA	0.932	0.904	0.887	0.977	0.910	0.904	0.904
HPM	0.932	0.910	0.887	0.989	0.915	0.898	0.887
BPM	0.926	0.910	0.887	0.977	0.910	0.904	0.898

**FIGURE 3:**
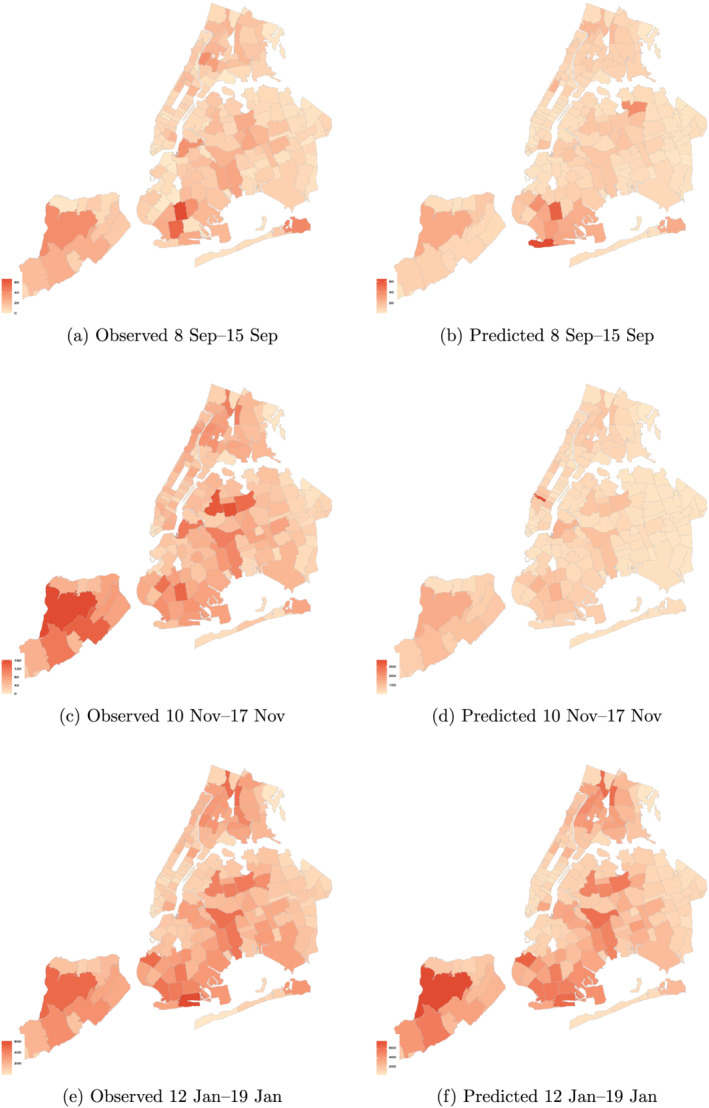
Predictive performance: Choropleth maps of observed and predicted (BPM) weekly new confirmed COVID‐19 cases in selected periods

To illustrate further the predictive ability of our proposed framework we show the evolution of the pandemic graphically. With this aim, Figure 3 presents choropleth maps for the modified ZCTAs of NYC comparing, for three selected weeks, the observed number of new confirmed COVID‐19 cases (left panel) with the predicted number by the BPM estimator (right panel). In line with the results reported in tables 5 and  6, the maps show a strong similarity between the predictions and the observed values in most neighborhoods and periods. Interestingly, and although the model does not catch up with the rapid increase in the number of cases that took place in mid‐October, it replicates the cross‐sectional differences across neighborhoods. In contrast, the predictive framework works quite well at the end of the sample period, when the model captures both the momentum in the number of cases and the cross‐sectional variability.

## 5 CONCLUSION

We present a model based on Poisson point processes to study the evolution of the coronavirus pandemic in the city of New York. Our study makes use of information on the number of confirmed COVID‐19 cases at the zip code level from September 2020 to February 2021. The aim of the study has been twofold. First, we are interested in determining the demographic, socioeconomic, and spatial factors that have ability to explain the differences in the number of new confirmed cases across neighborhoods. Second, we have assessed the in‐sample and out‐of‐sample predictive performance of alternative models. To do this, we consider a benchmark given by a Poisson random variable, modeling the intensity rate of the increase in weekly confirmed cases, that is extended in several dimensions by (i) adding a large set of regressors containing autoregressive components, exogenous covariates, and spatial effects; and (ii) controlling for model uncertainty by averaging the predictions of all possible combinations of regressors using a Bayesian approach.

Our results show that the proposed predictive framework displays a good performance both in‐sample and out‐of‐sample during the second wave of the coronavirus pandemic in NYC. Importantly, the main predictors are the autoregressive component of the model given by the first lag of the incidence rate, population size, age‐related variables, and spatial effects capturing spillovers between neighborhoods. The shares of employment in the transport, education and health, and entertainment sectors also exhibit predictive ability. These results contrast with those findings for the first wave reported in the related literature that highlight the role of occupational variables, household size, and income.

The associated prediction intervals obtained from estimates of the mean square error and mean square prediction error display very good coverage probabilities, allowing us to construct interval forecasts. The econometric methodology proposed in this paper, and the insights obtained from the empirical study, can be applied as useful tools for policy evaluation to assess the effectiveness of specific policies such as social distancing measures, the use of face masks, and other non‐pharmaceutical interventions implemented at neighbourhood or regional level. Our cross‐sectional analysis and prediction exercise would allow policy makers with information on these measures to gauge their impact across regions and over time.

## Supporting information

PIRS_12615_OnlineAppendixPIRS_final.pdf

## Footnotes

1 Another related literature is concerned with the actual number of infections, suggesting that confirmed COVID‐19 cases may be only the tip of the iceberg. Hortacscu et al. (2021) and Manski and Molinari (2021) have developed approaches to estimating (or at least bounding) the number of unreported cases. Although we do not pursue this strategy in the present study, it is worth noting that our prediction models might be applied in this setting under appropriate inference methods to obtain insights into the extent of the actual rate of infections in a given geographical area.

2
https://github.com/nychealth/coronavirus-data/

3 This row‐standardization implies that the contribution of the incidence rate from contiguous neighborhoods to central neighborhoods is smaller than in neighborhoods that share borders with just a few other neighborhoods.

4 As noted before, we report the results on a 3‐week basis for the sake of clarity and due to space constraints. The full set of results for the period from 1 September 2020 to 2 February 2021 are included in the online appendix.

5 These methods have been implemented using the BAS R package (Clyde, 2020; The R Core Team, 2021).

6 Our empirical results are not very sensitive to the choice of the prior. The robustness check is available from the authors upon request.
