# Supplementary material for: Modeling the spread of COVID‐19 in New York City
Source: Pap Reg Sci. 2021 Jun 28;100(5):1209–29. doi: 10.1111/pirs.12615 (PMC8242800; doi:10.1111/pirs.12615)
Supplement: Supplementary file 1 — PIRS_12615_OnlineAppendixPIRS_final.pdf [file PIRS-100-1209-s001.pdf]

Modeling the spread of COVID-19 in New York City
ONLINE APPENDIX

Table A1: Weekly new confirmed COVID-19 cases in modified zip code tabulation areas (ZCTAs, N=177): Descriptive statistics.

Period	Total	Mean	Standard deviation	Minimum	Maximum
1 Sep-8 Sep	1,571	8.876	8.241	0	47
8 Sep-15 Sep	1,992	11.254	10.251	0	66
15 Sep-22 Sep	2,298	12.983	18.767	0	193
22 Sep-29 Sep	2,491	14.073	20.423	0	159
29 Sep-6 Oct	3,520	19.887	25.033	0	187
6 Oct-13 Oct	3,512	19.842	20.208	0	141
13 Oct-20 Oct	3,456	19.525	17.144	0	111
20 Oct-27 Oct	3,891	21.983	16.340	0	79
27 Oct-3 Nov	4,402	24.870	18.936	0	114
3 Nov-10 Nov	5,748	32.475	23.943	0	125
10 Nov-17 Nov	8,533	48.209	32.627	0	164
17 Nov-24 Nov	10,121	57.181	37.059	3	187
24 Nov-1 Dec	12,646	71.446	47.201	2	259
1 Dec-8 Dec	10,277	58.062	63.942	0	400
8 Dec-15 Dec	18,702	105.661	71.888	3	387
15 Dec-22 Dec	18,701	105.655	73.452	6	375
22 Dec-29 Dec	22,123	124.989	87.499	6	435
29 Dec-5 Jan	25,846	146.023	102.699	1	530
5 Jan-12 Jan	32,516	183.706	124.069	7	588
12 Jan-19 Jan	33,392	188.655	126.137	3	624
19 Jan-26 Jan	29,952	169.220	114.293	2	530
26 Jan-2 Feb	28,232	159.503	108.471	4	472

Table A2: Poisson regression: Weekly new confirmed COVID-19 cases.

	8 Sep-15 Sep	15 Sep-22 Sep	22 Sep-29 Sep	29 Sep-6 Oct	6 Oct-13 Oct	13 Oct-20 Oct	20 Oct-27 Oct	27 Oct-3 Nov	3 Nov-10 Nov	10 Nov-17 Nov	17 Nov-24 Nov	24 Nov-01 Dec	1 Dec-8 Dec	8 Dec-15 Dec	15 Dec-22 Dec	22 Dec-29 Dec	29 Dec-5 Jan	5 Jan-12 Jan	12 Jan-19 Jan	19 Jan-26 Jan	26 Jan-2 Feb
lcases	0.033***	0.029***	-0.017***	-0.005*	-0.006***	0.003	0.003	0.003	0.002	0.005***	3.01e-04	0.005***	0.005***	0.001***	3.71e-04	0.003***	0.001***	2.06e-04	3.46e-04*	2.18e-04	2.37e-04
	(0.007)	(0.005)	(0.004)	(0.003)	(0.002)	(0.003)	(0.003)	(0.004)	(0.002)	(0.001)	(0.001)	(0.001)	(5.02e-04)	(2.38e-04)	(3.55e-04)	(2.91e-04)	(2.62e-04)	(2.08e-04)	(1.76e-04)	(2.30e-04)	(2.81e-04)
lincid	-0.004	0.004	0.020***	0.014***	0.010***	0.006***	0.002	0.009***	0.003**	0.001***	0.004***	3.37e-04	0.001***	-3.24e-04**	0.001***	-3.74e-05	0.001***	0.001***	0.001***	0.001***	0.001***
	(0.004)	(0.003)	(0.003)	(0.002)	(0.002)	(0.002)	(0.002)	(0.002)	(0.001)	(3.54e-04)	(0.001)	(3.17e-04)	(02.62e-04)	(1.49e-04)	(2.45e-04)	(1.50e-04)	(1.79e-04)	(1.41e-04)	(1.18e-04)	(1.44e-04)	(1.90e-04)
lwcases	-0.009	0.024***	0.019***	0.002	-3.68e-04	0.013***	0.008***	-0.004	0.004**	-0.002**	0.004***	0.002***	0.003***	0.002***	0.001***	0.001***	0.001***	0.001***	3.78e-04***	2.02e-04*	0.001***
	(0.008)	(0.005)	(0.005)	(0.004)	(0.003)	(0.003)	(0.002)	(0.003)	(0.002)	(0.001)	(0.001)	(4.99e-04)	(4.43e-04)	(3.30e-04)	(2.31e-04)	(1.85e-04)	(1.57e-04)	(1.31e-04)	(1.03e-04)	(1.05e-04)	(1.27e-04)
lwincid	0.021***	-0.002	-0.015***	0.001	0.003	-0.010***	-0.007**	0.012***	0.006***	0.005***	0.002***	0.002***	4.09e-04	4.28e-04**	0.001***	4.63e-04***	0.001***	2.18e-04*	2.66e-04**	0.001***	4.70e-04***
	(0.006)	(0.003)	(0.005)	(0.004)	(0.002)	(0.003)	(0.003)	(0.002)	(0.002)	(0.001)	(0.001)	(3.07e-04)	(2.86e-04)	(1.78e-04)	(2.26e-04)	(1.43e-04)	(1.55e-04)	(1.16e-04)	(1.06e-04)	(1.43e-04)	(1.75e-04)
popul	1.49e-05***	4.32e-06	1.50e-05***	1.38e-05***	1.50e-05***	9.45e-06***	1.75e-05***	1.15e-05***	1.56e-05***	1.15e-05***	1.31e-05***	6.67e-06***	9.91e-06***	1.03e-05***	1.13e-05***	7.96e-06***	1.10e-05***	1.30e-05***	1.18e-05***	1.29e-05***	1.11e-05***
	(3.03e-06)	(3.00e-06)	(2.85e-06)	(2.44e-06)	(2.35e-06)	(2.46e-06)	(2.28e-06)	(2.57e-06)	(2.14e-06)	(1.54e-06)	(1.50e-06)	(1.26e-06)	(1.40e-06)	(9.45e-07)	(1.15e-06)	(9.27e-07)	(9.11e-07)	(9.20e-07)	(9.51e-07)	(1.20e-06)	(1.32e-06)
hhsize	-0.839***	0.065	-0.041	-0.176	-0.198*	-0.177	-0.322***	-0.288***	-0.234***	-0.279***	-0.183***	-0.092	-0.583***	0.066	0.039	-0.066	0.044	0.071**	-0.096***	-0.075**	-0.069*
	(0.148)	(0.150)	(0.137)	(0.114)	(0.110)	(0.111)	(0.104)	(0.096)	(0.084)	(0.074)	(0.067)	(0.060)	(0.068)	(0.050)	(0.047)	(0.044)	(0.041)	(0.036)	(0.035)	(0.037)	(0.037)
male	-0.041**	-0.012	-0.002	-0.014	-0.004	0.027**	0.018*	0.001	0.011	-0.012	-0.004	-0.011*	0.035***	-0.025***	-0.012**	-0.005	-0.024***	-0.007*	-0.002	-0.006	-0.018***
	(0.017)	(0.016)	(0.017)	(0.013)	(0.014)	(0.012)	(0.011)	(0.011)	(0.009)	(0.008)	(0.007)	(0.006)	(0.007)	(0.005)	(0.005)	(0.005)	(0.005)	(0.004)	(0.004)	(0.004)	(0.004)
black	-0.003	-0.004	-0.010***	-0.006***	-0.003	-0.006***	-0.008***	-0.002	-0.001	-0.006***	-2.66e-04	-0.003***	-0.015***	-0.003***	-0.004***	-0.005**	-0.004***	-0.003***	-0.001**	-0.003***	0.001*
	(0.002)	(0.002)	(0.002)	(0.002)	(0.002)	(0.002)	(0.002)	(0.002)	(0.001)	(0.001)	(0.001)	(0.001)	(0.001)	(0.001)	(0.001)	(0.001)	(0.001)	(0.001)	(0.001)	(0.001)	(0.001)
hispanic	0.006**	-0.003	-0.011***	-0.001	0.001	2.85e-04	0.001	0.006***	0.004***	0.004***	0.004***	0.004***	-0.005***	0.004***	-4.50e-04	0.001*	0.003***	0.001**	2.40e-04	0.002***	2.16e-04
	(0.002)	(0.002)	(0.002)	(0.002)	(0.002)	(0.002)	(0.002)	(0.002)	(0.001)	(0.001)	(0.001)	(0.001)	(0.001)	(0.001)	(0.001)	(0.001)	(0.001)	(0.001)	(5.49e-04)	(0.001)	(0.001)
age	-0.045***	-0.030**	-0.069***	-0.064***	-0.056***	-0.044***	-0.037***	-0.058***	-0.015*	-0.023***	-0.021***	-0.028***	-0.069***	-0.031***	-0.020***	-0.017***	-0.026***	-0.019***	-0.018***	-0.012***	-0.026***
	(0.014)	(0.014)	(0.013)	(0.011)	(0.011)	(0.011)	(0.010)	(0.009)	(0.009)	(0.007)	(0.006)	(0.005)	(0.006)	(0.005)	(0.005)	(0.004)	(0.004)	(0.004)	(0.003)	(0.004)	(0.004)
over64	-1.04e-05	5.33e-05***	6.64e-05***	4.49e-05***	5.63e-05***	5.31e-05***	2.56e-05*	4.63e-05***	1.43e-05	2.75e-05***	3.81e-05***	4.46e-05***	-6.55e-07	5.08e-05***	4.70e-05***	2.58e-05***	2.78e-05***	3.68e-05***	3.69e-05***	3.57e-05***	5.09e-05***
	(1.98e-05)	(1.91e-05)	(1.83e-05)	(1.59e-05)	(1.55e-05)	(1.53e-05)	(1.40e-05)	(1.31e-05)	(1.13e-05)	(9.02e-06)	(8.46e-06)	(7.68e-06)	(9.01e-06)	(6.53e-06)	(6.42e-06)	(6.28e-06)	(5.75e-06)	(4.84e-06)	(4.74e-06)	(4.92e-06)	(5.16e-06)
enroll	-0.002	0.001	0.015	-0.016*	-0.003	-0.011	-0.008	-0.011	0.008	-0.007	0.009*	-0.013***	-0.065***	-0.019***	-0.016***	-0.019***	-0.025***	-0.014***	-0.004	-0.015***	-0.012***
	(0.012)	(0.011)	(0.011)	(0.010)	(0.010)	(0.010)	(0.008)	(0.008)	(0.007)	(0.006)	(0.005)	(0.005)	(0.006)	(0.004)	(0.004)	(0.004)	(0.004)	(0.003)	(0.003)	(0.003)	(0.003)
income	-9.47e-06***	-4.41e-06	-5.64e-06*	-5.95e-06**	-9.85e-06***	6.94e-07	2.41e-06	1.69e-06	-3.73e-06**	-1.06e-06	3.87e-06***	-2.62e-06**	-2.23e-06	-2.81e-06***	1.37e-06	-2.24e-06**	-5.56e-07	2.61e-07	-1.05e-06	-4.27e-06***	-3.60e-06***
	(3.31e-06)	(3.13e-06)	(3.23e-06)	(2.62e-06)	(2.70e-06)	(2.51e-06)	(2.19e-06)	(2.14e-06)	(1.82e-06)	(1.51e-06)	(1.34e-06)	(1.24e-06)	(1.51e-06)	(1.07e-06)	(1.07e-06)	(1.01e-06)	(9.48e-07)	(8.24e-07)	(8.28e-07)	(8.75e-07)	(8.90e-07)
selfemp	0.045***	0.031***	0.014	0.029***	0.018**	0.024***	0.010	0.005	0.015**	0.034***	0.012**	0.012***	-0.011**	0.012***	0.008**	0.007**	0.007**	-0.002	0.010***	0.018***	4.99e-04
	(0.011)	(0.010)	(0.010)	(0.009)	(0.008)	(0.008)	(0.008)	(0.007)	(0.006)	(0.005)	(0.005)	(0.004)	(0.005)	(0.004)	(0.004)	(0.003)	(0.003)	(0.003)	(0.003)	(0.003)	(0.003)
empl	0.026	-0.018	0.015	0.027**	0.040***	0.021	-0.007	0.038***	0.021**	0.032***	-0.018**	0.002	-0.023***	-0.010**	-0.011**	-0.001	0.005	-0.010**	-0.004	-0.022***	-0.008*
	(0.016)	(0.016)	(0.016)	(0.013)	(0.012)	(0.013)	(0.012)	(0.011)	(0.010)	(0.008)	(0.007)	(0.006)	(0.007)	(0.005)	(0.005)	(0.005)	(0.004)	(0.004)	(0.0045)	(0.004)	(0.004)
fulltime	0.003	-0.015	-0.011	-0.003	-0.018**	0.011	-1.93e-04	-0.012*	-0.006	0.006	-0.001	-0.003	-0.007*	-0.007**	0.001	0.001	-0.001	0.001	0.002	0.010***	-0.011***
	(0.010)	(0.010)	(0.009)	(0.008)	(0.008)	(0.008)	(0.007)	(0.007)	(0.006)	(0.005)	(0.004)	(0.004)	(0.004)	(0.003)	(0.003)	(0.003)	(0.003)	(0.002)	(0.002)	(0.002)	(0.003)
pubtrans	-0.004	-0.010**	-0.009**	-0.006*	-0.009***	0.002	-0.006**	1.41e-04	-0.002	3.46e-04	-0.004**	-0.005***	0.022***	-0.009***	-0.003**	-2.42e-04	-0.002*	0.001	-0.003***	-0.001	-0.001
	(0.004)	(0.004)	(0.004)	(0.003)	(0.003)	(0.003)	(0.003)	(0.003)	(0.002)	(0.002)	(0.002)	(0.002)	(0.002)	(0.001)	(0.001)	(0.001)	(0.001)	(0.001)	(0.001)	(0.001)	(0.001)
travtime	-4.70e-06	-5.17e-05	1.53e-04*	6.16e-06	1.40e-04*	3.61e-05	-4.51e-05	1.14e-04*	-6.47e-05	3.30e-05	7.33e-05*	-1.23e-04***	-3.77e-04***	2.41e-05	5.35e-06	1.79e-05	2.78e-05	2.15e-05	6.99e-05***	-1.45e-04***	-1.45e-04***
	(9.38e-05)	(9.62e-05)	(8.84e-05)	(7.58e-05)	(7.25e-05)	(7.56e-05)	(7.17e-05)	(6.49e-05)	(5.74e-05)	(4.75e-05)	(4.28e-05)	(4.03e-05)	(4.85e-05)	(3.21e-05)	(3.35e-05)	(3.06e-05)	(2.87e-05)	(2.58e-05)	(2.46e-05)	(2.81e-05)	(2.69e-05)
retail	0.015	0.026	-9.70e-06	0.012	-0.001	0.037**	0.032**	-0.011	0.005	0.050***	-0.017**	0.009	0.018**	-0.007	0.016***	-0.001	0.022***	0.016***	0.003	0.024***	0.008
	(0.018)	(0.018)	(0.017)	(0.015)	(0.014)	(0.014)	(0.013)	(0.013)	(0.011)	(0.009)	(0.008)	(0.007)	(0.008)	(0.006)	(0.006)	(0.006)	(0.005)	(0.005)	(0.004)	(0.005)	(0.005)
transp	0.056***	0.036**	0.010	0.034**	0.046***	0.046***	0.066***	0.030**	0.021**	0.041***	0.030***	0.036***	0.132***	0.044***	0.034***	0.034***	0.032***	0.025***	0.039***	0.033***	0.026***
	(0.017)	(0.017)	(0.017)	(0.014)	(0.013)	(0.013)	(0.012)	(0.012)	(0.010)	(0.008)	(0.007)	(0.007)	(0.008)	(0.006)	(0.006)	(0.005)	(0.005)	(0.004)	(0.004)	(0.004)	(0.005)
eduheal	0.018*	0.014	0.018*	0.031***	-0.013*	0.034***	0.011	0.023***	0.005	0.025***	-0.005	0.015***	0.044***	-0.001	0.016***	0.013***	0.013***	0.002	0.006**	0.014***	-0.004
	(0.010)	(0.009)	(0.009)	(0.008)	(0.008)	(0.008)	(0.007)	(0.007)	(0.006)	(0.005)	(0.004)	(0.004)	(0.004)	(0.003)	(0.003)	(0.003)	(0.003)	(0.002)	(0.002)	(0.003)	(0.003)
entert	-0.016	0.050***	-0.010	0.036***	-0.002	0.028***	0.036***	0.006	0.017**	0.023***	0.027***	0.015***	-0.008	0.023***	0.031***	0.009**	0.017***	0.013***	0.015***	0.017***	0.009**
	(0.015)	(0.015)	(0.014)	(0.012)	(0.011)	(0.011)	(0.010)	(0.009)	(0.008)	(0.007)	(0.006)	(0.005)	(0.006)	(0.005)	(0.005)	(0.004)	(0.004)	(0.003)	(0.003)	(0.004)	(0.004)
occup	0.005	0.001	0.009	0.002	0.015**	-0.008	0.002	0.002	-0.002	-0.005	0.014***	-0.005*	-0.009**	0.002	-0.001	0.004	0.010***	0.008***	0.015***	-0.002	0.010***
	(0.008)	(0.007)	(0.007)	(0.006)	(0.006)	(0.006)	(0.005)	(0.005)	(0.004)	(0.003)	(0.003)	(0.003)	(0.004)	(0.003)	(0.002)	(0.003)	(0.002)	(0.002)	(0.002)	(0.002)	(0.002)
rooms	0.203***	-0.208***	-0.206***	0.039	-0.093	0.121**	0.103*	0.138***	0.036	0.157***	0.074**	0.067**	0.602***	0.007	-0.028	0.113***	0.081***	0.018	-0.023	0.042**	0.030
	(0.073)	(0.074)	(0.074)	(0.060)	(0.058)	(0.059)	(0.053)	(0.050)	(0.044)	(0.036)	(0.035)	(0.031)	(0.035)	(0.028)	(0.026)	(0.023)	(0.022)	(0.020)	(0.019)	(0.020)	(0.021)
internet	0.009	0.030***	-0.004	0.004	0.006	-0.002	-0.001	0.002	0.001	-0.009***	-0.005*	0.005*	-0.013***	0.001	-0.004*	-0.010***	-0.005***	-0.003	-0.002	-0.006***	0.008***
	(0.007)	(0.007)	(0.007)	(0.006)	(0.005)	(0.005)	(0.005)	(0.005)	(0.004)	(0.003)	(0.003)	(0.003)	(0.003)	(0.002)	(0.002)	(0.002)	(0.002)	(0.002)	(0.002)	(0.002)	(0.002)
nocov	-0.003	-0.029***	-0.011	-0.021***	-0.036***	-0.007	-0.018***	-0.002	-0.020***	-0.006	-0.020***	0.001	-0.016***	-0.007**	-0.008***	-0.001	-0.007***	-0.011***	-7.84e-06	-0.001	3.11e-04
	(0.010)	(0.010)	(0.009)	(0.007)	(0.007)	(0.007)	(0.007)	(0.006)	(0.005)	(0.004)	(0.004)	(0.004)	(0.004)	(0.003)	(0.003)	(0.003)	(0.002)	(0.002)	(0.002)	(0.002)	(0.002)
Constant	1.541	3.392*	3.314*	1.320	0.882	-1.678	2.843**	-0.618	0.801	-0.084	3.666***	4.659***	7.737***	7.195***	5.030***	4.516***	3.929***	4.461***	3.092***	6.111***	5.867***
	(1.950)	(1.910)	(1.902)	(1.602)	(1.574)	(1.517)	(1.372)	(1.327)	(1.129)	(0.921)	(0.831)	(0.760)	(0.871)	(0.652)	(0.644)	(0.606)	(0.558)	(0.477)	(0.468)	(0.489)	(0.514)
Log likelihood	-601.626	-598.804	-632.269	-589.701	-584.575	-576.792	-597.097	-598.093	-786.253	-764.797	-734.456	-870.767	-2,741.613	-940.975	-905.818	-926.591	-1,107.054	-1,165.981	-1,064.428	-1,148.809	-1,015.036

Note: The number of observations is 177. Standard errors in parentheses. *** pj0.01, ** pj0.05, * pj0.1.

Table A3: Bayesian model averaging: Posterior inclusion probabilities.

	8 Sep-15 Sep	15 Sep-22 Sep	22 Sep-29 Sep	29 Sep-06 Oct	6 Oct-13 Oct	13 Oct-20 Oct	20 Oct-27 Oct	27 Oct-3 Nov	3 Nov-10 Nov	10 Nov-17 Nov	17 Nov-24 Nov	24 Nov-1 Dec	1 Dec-8 Dec	8 Dec-15 Dec	15 Dec-22 Dec	22 Dec-29 Dec	29 Dec-5 Jan	5 Jan-12 Jan	12 Jan-19 Jan	19 Jan-26 Jan	26 Jan-2 Feb
lcases	0.274	1.000	1.000	1.000	0.133	0.873	0.064	0.767	0.075	0.090	1.000	0.040	1.000	1.000	1.000	0.055	1.000	0.820	0.067	0.227	0.039
lincid	0.999	0.045	0.143	1.000	1.000	1.000	0.993	0.277	0.999	0.980	0.385	1.000	0.103	0.982	0.542	1.000	0.056	1.000	1.000	1.000	1.000
lwcases	0.038	0.104	1.000	0.377	0.246	0.219	0.975	0.410	0.078	0.274	0.255	1.000	1.000	1.000	1.000	0.992	0.994	0.994	1.000	0.974	0.139
lwincid	0.040	0.986	0.040	0.244	0.224	0.624	0.809	0.373	1.000	1.000	1.000	0.849	1.000	0.067	0.304	1.000	0.836	1.000	0.543	0.498	1.000
popul	0.181	1.000	0.150	1.000	1.000	1.000	0.999	1.000	1.000	1.000	1.000	1.000	0.999	1.000	1.000	1.000	1.000	1.000	1.000	1.000	1.000
hhsize	0.158	1.000	0.061	0.053	0.070	0.095	0.084	0.758	0.127	0.141	0.996	0.077	0.105	1.000	0.396	0.055	0.140	0.137	0.292	0.950	0.092
male	0.066	0.159	0.057	0.043	0.038	0.045	0.959	0.245	0.043	0.128	0.045	0.054	0.053	1.000	0.999	0.244	0.060	0.999	0.162	0.043	0.047
black	0.990	0.298	0.168	1.000	0.965	0.997	0.638	1.000	0.088	0.467	1.000	0.046	0.808	1.000	1.000	1.000	1.000	1.000	0.982	0.836	0.997
hispanic	0.189	0.720	0.050	1.000	0.307	0.141	0.121	0.055	0.989	0.719	0.945	0.899	0.917	1.000	1.000	0.057	0.682	0.998	0.621	0.114	0.268
age	0.926	0.988	0.896	1.000	0.997	0.995	0.680	0.589	0.997	0.093	0.458	0.709	0.985	1.000	1.000	0.999	0.976	1.000	1.000	1.000	0.402
over64	0.999	0.060	0.939	0.992	0.931	0.982	0.846	0.166	0.987	0.076	0.423	0.991	1.000	0.040	1.000	1.000	0.964	1.000	1.000	1.000	1.000
enroll	0.044	0.060	0.054	0.091	0.135	0.051	0.121	0.057	0.080	0.272	0.056	0.375	0.475	1.000	0.987	0.973	1.000	1.000	0.990	0.096	0.990
income	0.993	0.583	0.277	0.894	0.970	0.990	0.084	0.060	0.048	0.910	0.049	0.835	0.218	0.081	0.906	0.214	0.640	0.045	0.046	0.126	1.000
selfemp	0.151	0.904	0.279	0.080	0.956	0.594	0.332	0.097	0.051	0.719	1.000	0.136	0.440	0.476	0.987	0.113	0.149	0.334	0.094	0.388	1.000
empl	0.045	0.110	0.053	0.037	0.349	0.628	0.095	0.117	0.901	0.152	0.958	0.850	0.042	0.992	0.578	0.676	0.050	0.051	0.909	0.126	1.000
fulltime	0.048	0.068	0.992	0.264	0.094	0.119	0.139	0.081	0.047	0.246	0.114	0.075	0.083	0.074	0.495	0.149	0.049	0.149	0.061	0.046	1.000
pubtrans	0.122	0.096	0.275	0.072	0.170	0.147	0.105	0.411	0.055	0.064	0.053	0.304	0.767	1.000	1.000	0.571	0.080	0.169	0.046	0.465	0.048
travtime	0.047	0.059	0.072	0.109	0.045	0.133	0.066	0.108	0.075	0.749	0.050	0.049	0.832	1.000	0.194	0.110	0.065	0.052	0.164	0.956	1.000
retail	0.041	0.046	0.144	0.041	0.042	0.039	0.164	0.622	0.068	0.039	1.000	0.150	0.042	0.559	0.047	0.475	0.041	0.996	0.985	0.058	1.000
transp	0.049	0.892	0.527	0.074	0.121	0.985	0.699	0.998	0.063	0.052	1.000	0.868	0.998	1.000	1.000	1.000	1.000	1.000	1.000	1.000	1.000
eduheal	0.093	0.500	0.060	0.998	0.952	0.065	0.862	0.121	0.993	0.089	1.000	0.079	0.832	1.000	0.049	0.998	0.999	1.000	0.058	0.510	1.000
entert	0.052	0.470	0.701	0.083	0.127	0.057	0.261	0.954	0.043	0.347	0.990	0.989	0.861	0.045	1.000	1.000	0.528	0.999	1.000	0.999	0.999
occup	0.038	0.173	0.044	0.095	0.050	0.568	0.047	0.087	0.080	0.040	0.067	1.000	0.122	0.538	0.134	0.043	0.549	0.999	1.000	1.000	0.036
rooms	0.055	0.989	0.224	0.335	0.084	0.110	0.102	0.240	0.455	0.049	0.994	0.158	0.359	1.000	0.190	0.087	1.000	0.996	0.092	0.132	0.073
internet	0.041	0.162	0.998	0.147	0.088	0.049	0.078	0.047	0.129	0.065	0.377	0.098	0.339	1.000	0.091	0.266	1.000	0.738	0.051	0.082	0.987
nocov	0.052	0.065	0.567	0.043	0.730	1.000	0.095	0.927	0.098	0.996	0.130	1.000	0.057	0.999	0.731	0.462	0.047	0.127	1.000	0.045	0.045

Note: The number of observations is 177. The sampling method consists of a random walk Metropolist-Hastings algorithm combined with a random swap. A benchmark and uniform priors have been established, respectively, for parameters and models.

Table A4: Predictive performance: Root mean square errors.

	8 Sep-15 Sep	15 Sep-22 Sep	22 Sep-29 Sep	29 Sep-6 Oct	6 Oct-13 Oct	13 Oct-20 Oct	20 Oct-27 Oct	27 Oct-3 Nov	3 Nov-10 Nov	10 Nov-17 Nov	17 Nov-24 Nov	24 Nov-1 Dec	1 Dec-8 Dec	8 Dec-15 Dec	15 Dec-22 Dec	22 Dec-29 Dec	29 Dec-5 Jan	5 Jan-12 Jan	12 Jan-19 Jan	19 Jan-26 Jan
Relative, in-sample																				
Benchmark	542.735	504.081	582.623	942.967	699.926	522.601	766.300	751.601	1044.899	1136.260	805.403	558.350	637.715	2082.188	661.461	565.335	669.731	2557.772	602.540	900.170
Poisson	1.068	1.208	1.042	1.094	0.993	0.720	0.886	0.817	0.976	1.064	0.772	0.369	1.145	2.313	0.630	0.364	0.506	1.456	0.558	0.851
BMA	1.080	1.205	1.077	1.205	1.013	0.704	0.951	0.989	1.033	1.147	0.826	0.400	1.206	2.246	0.620	0.381	0.532	1.486	0.538	0.862
HPM	1.093	1.226	1.063	1.243	1.020	0.718	0.928	1.003	1.048	1.149	0.858	0.398	1.204	2.272	0.628	0.386	0.547	1.512	0.530	0.870
BPM	1.102	1.211	1.066	1.213	1.010	0.693	0.922	1.007	1.042	1.141	0.848	0.396	1.224	2.285	0.610	0.377	0.525	1.512	0.535	0.869
Relative, out-of-sample																				
	8 Sep-15 Sep	15 Sep-22 Sep	22 Sep-29 Sep	29 Sep-6 Oct	6 Oct-13 Oct	13 Oct-20 Oct	20 Oct-27 Oct	27 Oct-3 Nov	3 Nov-10 Nov	10 Nov-17 Nov	17 Nov-24 Nov	24 Nov-1 Dec	1 Dec-8 Dec	8 Dec-15 Dec	15 Dec-22 Dec	22 Dec-29 Dec	29 Dec-5 Jan	5 Jan-12 Jan	12 Jan-19 Jan	19 Jan-26 Jan
Benchmark	495.856	604.039	589.974	509.491	618.790	500.883	708.343	682.313	498.974	385.556	556.704	1120.388	414.464	372.782	388.984	1982.334	442.216	505.478	1184.900	532.442
Poisson	1.326	1.281	8.955	0.624	0.875	1.000	0.672	0.974	0.835	0.697	0.495	1.117	4.145	0.707	0.518	0.558	1.435	0.424	1.208	1.122
BMA	1.380	1.297	10.405	0.712	0.842	0.980	0.848	1.146	0.884	1.670	0.521	1.200	4.197	0.710	0.509	0.614	1.550	0.425	1.166	1.226
HPM	1.420	1.338	11.724	0.729	0.827	0.976	0.813	1.127	0.895	2.250	0.542	1.235	4.142	0.705	0.513	0.610	1.565	0.430	1.166	1.215
BPM	1.399	1.338	11.440	0.731	0.835	0.974	0.804	1.104	0.886	1.572	0.527	1.187	4.241	0.716	0.505	0.604	1.562	0.430	1.166	1.217
Absolute, in-sample																				
	8 Sep-15 Sep	15 Sep-22 Sep	22 Sep-29 Sep	29 Sep-6 Oct	6 Oct-13 Oct	13 Oct-20 Oct	20 Oct-27 Oct	27 Oct-3 Nov	3 Nov-10 Nov	10 Nov-17 Nov	17 Nov-24 Nov	24 Nov-1 Dec	1 Dec-8 Dec	8 Dec-15 Dec	15 Dec-22 Dec	22 Dec-29 Dec	29 Dec-5 Jan	5 Jan-12 Jan	12 Jan-19 Jan	19 Jan-26 Jan
Benchmark	10.251	18.767	20.423	25.033	20.208	17.144	16.340	18.936	23.943	32.627	37.059	47.201	63.942	71.888	73.452	87.499	102.699	124.069	126.137	114.293
Poisson	4.781	5.781	8.675	7.629	6.518	6.906	6.365	6.710	6.719	10.948	12.206	12.885	16.622	36.160	21.232	21.571	21.801	28.649	31.966	29.285
BMA	4.829	5.983	8.850	7.900	6.747	7.163	6.507	6.708	7.149	11.190	12.341	13.103	16.545	35.975	21.191	21.763	21.728	*28.835*	32.423	*29.547*
HPM	4.889	6.002	8.646	7.989	6.743	7.192	6.530	6.891	7.315	11.320	12.476	13.241	16.697	36.117	21.118	21.983	21.876	28.876	32.744	29.555
BPM	4.872	6.004	8.594	7.986	6.721	7.081	6.548	6.863	7.273	11.208	12.487	13.210	16.842	36.054	21.246	21.981	21.801	28.876	32.662	29.652
Absolute, out-of-sample																				
	8 Sep-15 Sep	15 Sep-22 Sep	22 Sep-29 Sep	29 Sep-6 Oct	6 Oct-13 Oct	13 Oct-20 Oct	20 Oct-27 Oct	27 Oct-3 Nov	3 Nov-10 Nov	10 Nov-17 Nov	17 Nov-24 Nov	24 Nov-1 Dec	1 Dec-8 Dec	8 Dec-15 Dec	15 Dec-22 Dec	22 Dec-29 Dec	29 Dec-5 Jan	5 Jan-12 Jan	12 Jan-19 Jan	19 Jan-26 Jan
Benchmark	10.396	18.799	21.234	25.033	20.210	17.319	16.593	20.406	28.650	33.838	39.710	49.062	79.714	71.888	75.954	89.992	109.394	124.168	127.625	114.705
Poisson	8.907	13.850	13.579	10.101	23.319	7.661	9.320	9.565	15.643	17.926	27.999	24.656	59.494	90.221	50.137	34.309	39.484	46.244	55.450	48.870
BMA	9.248	13.104	15.824	10.013	23.196	7.273	9.274	9.695	14.813	33.520	30.267	25.234	60.378	91.751	49.836	34.886	39.502	45.824	55.294	49.002
HPM	9.286	13.173	17.879	10.147	23.163	7.379	9.337	10.057	14.786	44.262	32.254	26.109	59.464	91.594	51.467	34.768	39.506	45.707	56.040	48.588
BPM	9.178	13.236	17.441	10.299	23.174	7.266	9.317	9.711	14.692	31.931	30.360	24.745	60.705	91.091	50.664	34.740	39.256	45.708	55.403	48.572

Table A5: Predictive performance: Empirical coverage probabilities.

In-sample	8 Sep-15 Sep	15 Sep-22 Sep	22 Sep-29 Sep	29 Sep-6 Oct	6 Oct-13 Oct	13 Oct-20 Oct	20 Oct-27 Oct	27 Oct-3 Nov	3 Nov-10 Nov	10 Nov-17 Nov	17 Nov-24 Nov	24 Nov-1 Dec	1 Dec-8 Dec	8 Dec-15 Dec	15 Dec-22 Dec	22 Dec-29 Dec	29 Dec-5 Jan	5 Jan-12 Jan	12 Jan-19 Jan	19 Jan-26 Jan
Benchmark	0.96	0.989	0.972	0.960	0.938	0.932	0.938	0.949	0.966	0.977	0.949	0.977	0.96	0.977	0.938	0.955	0.949	0.972	0.932	0.893
Poisson	0.915	0.904	0.955	0.926	0.898	0.893	0.904	0.898	0.904	0.910	0.904	0.898	0.915	0.915	0.887	0.915	0.910	0.904	0.887	0.926
BMA	0.926	0.904	0.943	0.910	0.887	0.910	0.910	0.910	0.898	0.904	0.898	0.881	0.910	0.915	0.898	0.915	0.910	0.910	0.887	0.904
HPM	0.921	0.904	0.943	0.915	0.893	0.893	0.915	0.915	0.898	0.898	0.904	0.870	0.910	0.921	0.893	0.921	0.926	0.910	0.881	0.893
BPM	0.921	0.904	0.938	0.910	0.898	0.904	0.910	0.904	0.898	0.915	0.898	0.893	0.921	0.921	0.887	0.932	0.91	0.91	0.893	0.898

Out-of-sample	8 Sep-15 Sep	15 Sep-22 Sep	22 Sep-29 Sep	29 Sep-6 Oct	6 Oct-13 Oct	13 Oct-20 Oct	20 Oct-27 Oct	27 Oct-3 Nov	3 Nov-10 Nov	10 Nov-17 Nov	17 Nov-24 Nov	24 Nov-1 Dec	1 Dec-8 Dec	8 Dec-15 Dec	15 Dec-22 Dec	22 Dec-29 Dec	29 Dec-5 Jan	5 Jan-12 Jan	12 Jan-19 Jan	19 Jan-26 Jan
Benchmark	0.949	0.966	0.949	0.943	0.943	0.938	0.915	0.949	0.949	0.932	0.955	0.949	0.955	0.932	0.932	0.926	0.932	0.932	0.921	0.926
Poisson	0.938	0.955	0.989	0.915	0.983	0.887	0.887	0.915	0.904	0.932	0.966	0.932	0.910	0.898	0.898	0.904	0.893	0.910	0.910	0.915
BMA	0.932	0.955	0.989	0.904	0.983	0.887	0.887	0.910	0.898	0.977	0.972	0.932	0.910	0.904	0.904	0.904	0.898	0.915	0.904	0.921
HPM	0.932	0.955	0.994	0.910	0.983	0.887	0.887	0.915	0.904	0.989	0.972	0.926	0.915	0.904	0.904	0.898	0.898	0.915	0.887	0.921
BPM	0.926	0.955	0.994	0.910	0.983	0.893	0.887	0.915	0.898	0.977	0.966	0.932	0.91	0.904	0.893	0.904	0.898	0.915	0.898	0.791
